# Long non-coding RNA LINRIS is upregulated in non-small cell lung cancer and its silencing inhibits cell proliferation by suppressing microRNA-10a maturation

**DOI:** 10.1080/21655979.2022.2031672

**Published:** 2022-02-09

**Authors:** Yajie Zhu, Ke Ma, Yingxue Ye, Jianning Tang, Jiang Zhu

**Affiliations:** a Department of Internal Medicine-Oncology, Sichuan Cancer Hospital, Chengdu City, Sichuan Province, China; b Department of Thoracic Surgery, Sichuan Cancer Hospital, Chengdu City, Sichuan Province, China

**Keywords:** Non-small cell lung cancer, LINRIS, miR-10a, maturation, proliferation

## Abstract

Long noncoding RNA LINRIS (LINC00920) is known to participate in colorectal cancer. This study aimed to explore the role of LINRIS in non-small cell lung cancer (NSCLC). NSCLC and adjacent non-tumor tissues were collected from 62 NSCLC patients. LINRIS expression was detected using real-time quantitative PCR (RT-qPCRs). The 62 NSCLC patients were monitored every month for 5 years to evaluate the role of LINRIS in predicting the prognosis of NSCLC. The effects of LINRIS silencing on microRNA-10a (miR-10a) precursor and mature miR-10a levels were assessed by RT-qPCR. Cell proliferation was measured using Cell Counting Kit-8 (CCK-8) assays. LINRIS expression was upregulated in NSCLC tissues. High LINRIS levels predicted poor survival of NSCLC patients. LINRIS were positively correlated with mature (miR-10a) levels but not miR-10a precursor. In NSCLC cells, LINRIS silencing showed no role in miR-10a precursor accumulation but downregulated mature miR10a level. Moreover, LINRIS silencing inhibited cell proliferation, while miR-10a overexpression increased cell proliferation and inhibited the role of LINRIS silencing. Overall, LINRIS silencing may inhibit NSCLC cell proliferation by suppressing miR-10a maturation.

**Abbreviations:** Non-small cell lung cancer (NSCLC); Reverse transcriptase-quantitative polymerase chain reaction (RT-qPCR); LncRNA long intergenic noncoding RNA for IGF2BP2 stability (LINRIS).

## Introduction

In clinical practice, lung cancer is the most frequently diagnosed cancer and is responsible for a considerable portion of cancer deaths [1]. It has been reported that lung cancer accounted for 11.6% and 18.4% of new cases and cancer deaths, respectively, in 2018 [2]. Non-small cell lung cancer (NSCLC) is the major subtype of lung cancer and accounts for about 85% of all lung cancer cases [3]. The most common risk factor for NSCLC is smoking. However, quitting smoking requires intensive life interferences and is not practical for all cases [4,5]. In addition, NSCLC affects never-smokers as well [6,7]. Therefore, novel preventative and treatment approaches are needed.

Target therapy aims to suppress cancer progression by regulating gene expression [8,9]. The development of target therapies requires a better understanding of the molecular mechanisms of cancer [10]. Alterations of molecular signaling pathways have been observed in NSCLC progression [11]. Long noncoding RNAs (LncRNAs) and micro RNAs (miR) play critical roles in cancers by regulating gene expression, suggesting their potential roles as targets to treat cancers [12,13]. However, the functions of most lncRNAs in cancer biology remain unclear. LncRNA LINRIS plays an oncogenic role in colorectal cancer [14]. MiR-10a promotes malignant phenotypes of various human cancers, such as lung cancer [15]. Therefore, this study was carried out to investigate the role of LINRIS in NSCLC and explore the crosstalk between LINRIS and miR-10a in NSCLC. We found that LINRIS silencing may inhibit NSCLC cell proliferation by suppressing miR-10a maturation.

## Methods

### NSCLC patients

A total of 62 NSCLC patients (42 males and 20 females) who underwent surgical resection at Sichuan Cancer Hospital from January 2013 to January 2015 were enrolled after the Ethics Committee of this hospital approved this study (No.1100120200614). All patients were newly diagnosed patients and recurrent NSCLC patients were excluded. The 62 patients were at the age of 50.1±± 6.2 years old on average in the range from 43 to 67 and included 28 cases of lung adenocarcinoma (LUAD) and 34 cases of squamous cell carcinoma (LUSC). All patients did not receive any chemotherapy, radiotherapy, targeted therapy, or immune therapy before surgery. Moreover, NSCLC patients with other synchronous malignancies were excluded. All patients signed written informed consent. All experiments were conducted in accordance with the Declaration of Helsinki.

### Follow-up

The 62 NSCLC patients included 26 cases at stage I–II and 36 cases at stage III–IV. Treatment strategies, such as chemotherapy, radiotherapy, surgery, or their combinations were performed. All the 62 patients were visited every month to record their survival. The patients died of NSCLC or completed the follow-up.

### NSCLC tissues and cells

NSCLC tumor and paired non-tumor tissue samples were collected from all patients. Tumor tissues were obtained from the central part of the lesion. The adjacent non-tumor tissues were more than 5 cm away from the tumor lesion. Non-tumor tissues were macroscopically and microscopically confirmed to be not invaded by cancer cells. Two NSCLC cell lines HCC4006 (LUAD) and NCI-H1703 (LUSC) (ATCC, USA) and normal human lung epithelial cells BEAS-2B (SCSP-5067, Cell Bank of Type Culture Collection of Chinese Academy of Sciences, Shanghai, China) were used in the study and cultured in media containing 10% FBS and 90% DMEM (GIBCO, California, USA) at 37°C in a humidified incubator with 5% CO_2_.

### Cell transfections

5 x 10^7^ cells were transfected with 40 nM LINRIS siRNA (Invitrogen, California, USA) or miR-10a mimic (10 nM) using Lipofectamine 2000 (Invitrogen, California, USA) [16]. Negative control (NC) miRNA or NC siRNA transfected cells were used as NC cells. After transfection, cells were cultured in fresh media for 48 h prior to subsequent assays. The sequences of siRNA used in the experiments were sh- CTACATAAAGCAGCCAATA (siLINRIS#1), CCATTTGGATCAGCTAATA (siLINRIS#2), and TTCTCCGAACGTGTCACGT (siNC).

### RNA preparation

Total RNAs were isolated from HCC4006 and NCI-H1703 cells and tissue samples using Trizol (Invitrogen, Carlsbad, CA). OD260/280 ratios of all RNA samples were determined to reflect the purity of RNA samples.

### RT-qPCR

RNA samples with OD260/280 ratio close to 2.0 were reverse transcribed (RT) into cDNA samples usin SSRT IV system (Invitrogen, California, USA). With cDNA samples as the template, qPCRs were performed with 18S rRNA as the internal control to study LINRIS expression [17]. The levels of mature miR-10a or miR-10a precursor were determined using RT-qPCRs. To analyze mature miR-10a, RT-qPCR was performed after poly(A) addition with U6 as the internal control using All-in-One™ miRNA qRT-PCR reagent kit (GeneCopoeia) with poly(T) primer for RTs and sequence-specific forward primer and poly(T) reverse primer for qPCRs. Ct values were normalized using the 2^−ΔΔCt^ method. The sequences of primers used in RT-qPCR assay were LINRIS forward 5’-ACTCTGCCTTTGGCTTTT-3’ and reverse 5’-ACTTTCACTCTTCCCTATGCT-3’; miR-10a precursor forward 5’-GGAAGGAGTCTTCGTGTGGC-3’ and reverse 5’-GCGCGGAAAGTAGGAGAACT-3’; GAPDH forward 5’-GGAGCGAGATCCCTCCAAAAT-3’ and reverse 5’-GGCTGTTGTCATACTTCTCATGG-3’; U6 forward 5’-GCTTCGGCAGCACATATACTAA-3’ and reverse 5’-TTGCGTGTCATCCTTGCG-3’.

### CCK-8 assay

Proliferation of HCC4006 and NCI-H1703 cells after transfections were detected using CCK-8 kits (Dojindo, Japan) [18]. After washed with PBS, 5000 cells were transferred to each well of a 96-well plate and cultured at 37°C. Cell numbers were reflected by measuring OD values of medium at 450 nm at 2 h after the addition of 10% CCK-8. Measurements were performed every 24 h for a total of 96 h.

### Statistical analyses

Paired t test was used to compare paired tissues. ANOVA Tukey’s test was used to compare data of 3 biological replicates of independent cell transfection groups. To analyze the role of LINRIS in predicting patients’ survival, the 62 patients were grouped into high and low LINRIS level groups (n = 31) with the median LINRIS level in NSCLC tissues as the cutoff value. Survival curves were plotted using the follow-up data and compared using log-rank test. *P* < 0.05 was considered statistically significant.

## Results

This study aimed to explore the role of LINRIS in NSCLC and explore the crosstalk between LINRIS and miR-10a in NSCLC. RT-qPCRs were performed to study LINRIS express in NSCLC tissues. The effects of LINRIS silencing on miR-10a precursor and mature miR-10a levels were assessed by RT-qPCR. Cell proliferation was determined by CCK-8 assay. LINRIS silencing might inhibit NSCLC cell proliferation by suppressing miR-10a maturation.

### LINRIS was upregulated in NSCLC tissues and correlated with poor survival of patients

**Figure 1.**
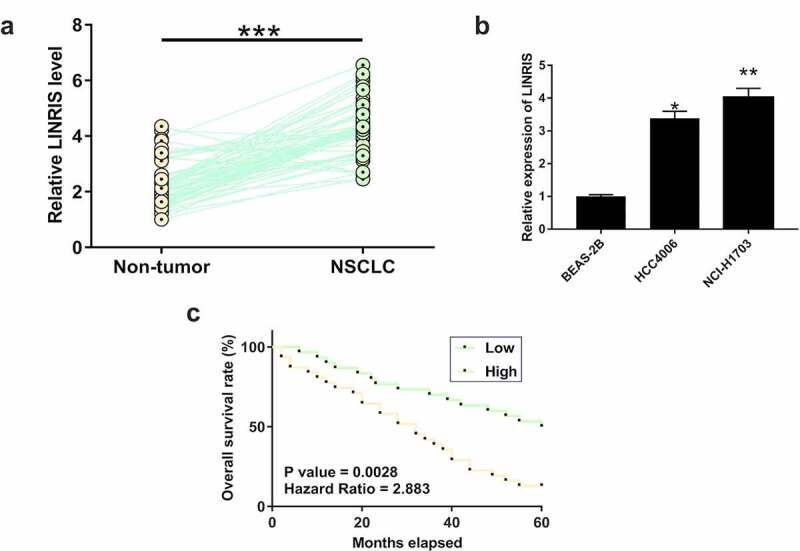
LINRIS upregulation predicted poor survival of NSCLC patients NSCLC and non-tumor tissues were collected from 62 NSCLC patients. RNA isolations and RT-qPCRs were performed to measure the expression levels of LINRIS (a). ***, *p* < 0.001. To perform survival analysis, the 62 patients were grouped into high and low LINRIS level groups (n = 31) with median LINRIS level in NSCLC tissues as the cutoff. Survival curves of both groups were plotted and compared using log rank test (b). LINRIS expression levels in NSCLC cell lines and normal lung epithelial cells (c). *, *p* < 0.05. **, *p* < 0.01.

Our deep sequencing data analysis revealed altered LINRIS expression in NSCLC (Figure S2). RT-qPCR showed that LINRIS expression levels were significantly higher in NSCLC tissues than in paired non-tumor tissues from the 62 NSCLC patients (Figure 1(a), *p* < 0.05). Consistently, LINRIS expression was markedly upregulated in NSCLC cell lines than in normal lung epithelial cells (Figure 1(b)). During the 5 years of follow-up, 42 out of the 62 patients died of NSCLC. Therefore, the 5-year overall survival rate of these patients was 32.3%, close to the overall 5-year survival rate (25%) of all stages of NSCLC patients worldwide [1–3]. By comparing the survival curves, we found that patients with high LINRIS levels exhibited significantly lower overall survival rates than patients with low LINRIS levels (Figure 1(c)). Therefore, LINRIS upregulation may predict poor survival of NSCLC patients.

### LINRIS was positively correlated with mature miR-10a across NSCLC tissues

**Figure 2.**
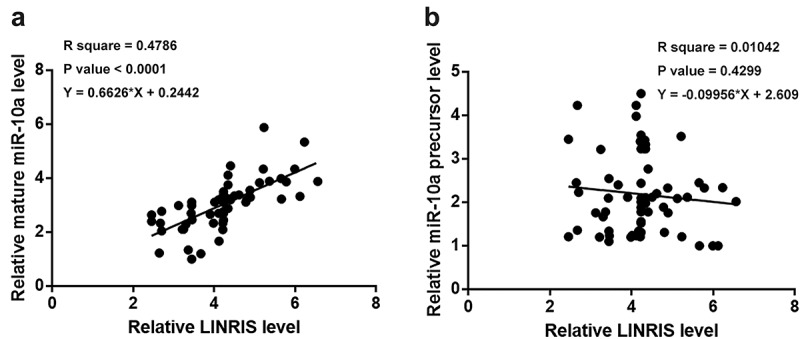
LINRIS was inversely correlated with mature miR-10a across NSCLC tissues The expression levels of mature miR-10a and miR-10a precursor were determined by RT-qPCRs. Correlations of LINRIS expression with mature miR-10a (a) or miR-10a precursor (b) were analyzed by linear regression.

The expression of mature miR-10a and miR-10a precursor were determined by RT-qPCR. Correlations of LINRIS with mature miR-10a (Figure 2(a)) and miR-10a precursor (Figure 2(b)) were analyzed by linear regression. It was observed that LINRIS expression levels were positively correlated with mature miR-10a levels across NSCLC tissues (r = 0.479, *p* < 0.001). Further subgroup analysis demonstrated a positive correlation between LINRIS expression and mature miR-10a levels, regardless of histological type or TNM stage (Fig. S1 A-D). In contrast, no significant correlation was observed between LINRIS expression and miR-10a precursor levels across NSCLC tissues.

### LINRIS silencing downregulated mature miR-10a in NSCLC cells

**Figure 3.**
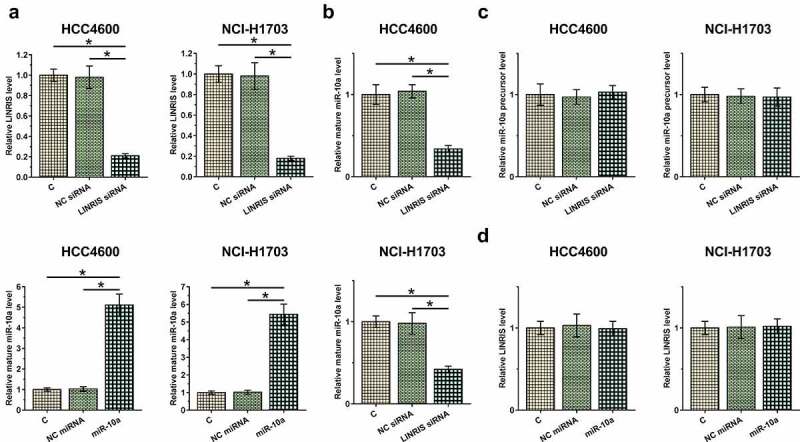
LINRIS silencing downregulated mature miR-10a in NSCLC cells HCC4006 and NCI-H1703 cells were transfected with LINRIS siRNA or miR-10a mimic to explore their relationship. Transfections were confirmed by RT-qPCRs (a). The effects of LINRIS silencing on the expression of miR-10a precursor (b) and mature miR-10a (c) and the effects of miR-10a mimic transfection on LINRIS expression (d) in both cell lines were analyzed by RT-qPCRs. *, *p* < 0.05.

HCC4006 and NCI-H1703 cells were transfected with LINRIS siRNA or miR-10a mimic to explore their relationship. Transfections were confirmed by RT-qPCR (Figure 3(a), *p* < 0.05). It was observed that LINRIS knockdown significantly decreased mature miR-10a level (Figure 3(b)), but it did not affect miR-10a precursor expression (Figure 3(c)). Moreover, miR-10a mimic transfection did not affect LINRIS expression in both cell lines (Figure 3(d)).

### LINRIS silencing inhibited NSCLC cell proliferation via miR-10a

**Figure 4.**
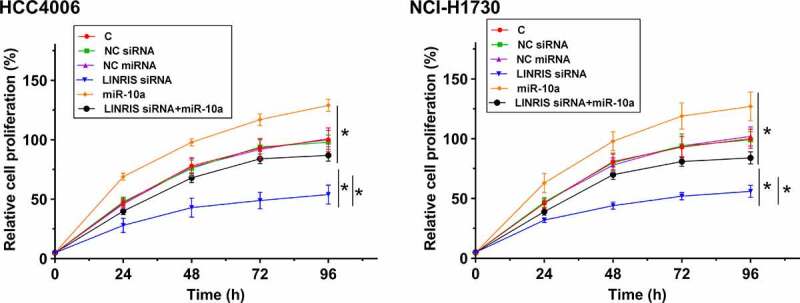
LINRIS silencing inhibited NSCLC cell proliferation via miR-10a The roles of LINRIS silencing and miR-10a overexpression in regulating the proliferation of HCC4006 and NCI-H1703 cells were analyzed by CCK-8 assay. *, *p* < 0.05.

The roles of LINRIS silencing and miR-10a overexpression in regulating the proliferation of HCC4006 and NCI-H1703 cells were assessed by CCK-8 assay. The results showed that LINRIS knockdown markedly inhibited the proliferation of HCC4006 and NCI-H1703 cells. In contrast, miR-10a overexpression promoted their proliferation (Figure 4). In addition, miR-10a overexpression partly reversed the inhibitory effect of LINRIS knockdown on NSCLC cell proliferation (Figure 4). Western blot analysis of cell cycle-related protein cyclin B showed that LINRIS knockdown significantly decreased cyclin B protein level in HCC4006 and NCI-H1703 cells while miR-10a overexpression increased cyclin B protein level in HCC4006 and NCI-H1703 cells (Figure S3A, B).

## Discussion

In this study, we investigated the involvement of LINRIS in NSCLC and explored its interaction with miR-10a. We found that LINRIS was upregulated in NSCLC and LINRIS silencing suppressed miR-10a maturation, resulting in the inhibition of NSCLC cell proliferation.

High aerobic glycolysis in cancer cells provides energy for cancer cell proliferation, migration, and invasion [19,20]. Wang *et al*. reported that LINRIS could promote aerobic glycolysis in colorectal cancer cells by stabilizing IGF2BP2 [14], suggesting an oncogenic role of LINRIS in colorectal cancer. We showed that LINRIS was upregulated in NSCLC. It has been well established that aerobic glycolysis is required for cell proliferation. In this study, we showed that LINRIS silencing decreased proliferation of both LUAD and LUSC cells, the two major subtypes of NSCLC. Therefore, LINRIS may play an oncogenic role in NSCLC by promoting cell proliferation and LINRIS silencing may serve as a potential therapeutic approach for NSCLC treatment.

Tumor metastasis is common in clinical practice [21]. The early diagnosis of NSCLC is unlikely to be significantly improved by discovering more biomarkers. We found that LINRIS expression was closely correlated with poor survival of NSCLC patients. Therefore, detecting LINRIS expression before therapies may assist the prognosis of NSCLC and improve the survival of patients by guiding the determination of treatments and care programs.

MiR-10a plays oncogenic roles in many human cancers, such as oral squamous cell carcinoma, pancreatic ductal adenocarcinoma, gastric cancer, and lung cancer [15,22–24]. Consistently, we showed that miR-10a overexpression increased NSCLC cell proliferation. It is reported that miR-10a could upregulate glucose transporter 1 (GLUT-1) to participate in glucose metabolism in oral squamous cell carcinoma [24]. In this study, we showed that LINRIS, a critical player in aerobic glycolysis, may regulate miR-10a maturation in NSCLC to regulate cell proliferation. It has been reported that lncRNAs are mainly involved in regulating gene expression by promoting miRNA stability or suppressing miRNA degradation [25]. We speculated that LINRIS might play an oncogenic role in the development and progression of NSCLC by regulating miR-10a stability. It is also possible that LINRIS may affect premature miR-10a transportation from the nucleus to the cytoplasm, which is required for miR-10a maturation in the cytoplasm, to affect mature miR-10a production. However, the underlying mechanism remains to be further elucidated. In addition, the interaction between LINRIS and miR-10a in glucose metabolism needs to be further investigated.

Previous studies showed that MiR-187 suppresses non-small-cell lung cancer cell proliferation by targeting FGF9 [26]. CircRNA_001846 may represent a promising diagnostic biomarker for NSCLC [27]. However, our deep sequencing data analysis revealed altered expression of LINRIS in NSCLC and investigated the crosstalk between LINRIS and miR-10a in NSCLC. We confirmed that silencing of LINRIS may inhibit NSCLC cell proliferation by suppressing the maturation of miR-10a.

Our study suggested that LINRIS downregulation could be targeted to suppress NSCLC growth by inhibiting miR-10a expression. However, clinical trials are needed to test our hypothesis. Moreover, this study is limited by the lack of animal model experiments and a small sample size. Our conclusions remain to be confirmed by future studies with *in vivo* assays and larger sample size.

## Conclusion

LINRIS is upregulated in NSCLC and predicts poor survival of NSCLC patients. Moreover, LINRIS silencing may suppress miR-10a maturation to inhibit NSCLC cell proliferation.

## Supplementary Material

Supplemental Material

## Data Availability

The datasets are available from the corresponding author on reasonable request.
