# Supplementary figures and images for: Long non-coding RNA LINRIS is upregulated in non-small cell lung cancer and its silencing inhibits cell proliferation by suppressing microRNA-10a maturation
Source: Bioengineered. 2022 Feb 9;13(2):4340–6. doi: 10.1080/21655979.2022.2031672 (PMC8973870; doi:10.1080/21655979.2022.2031672)

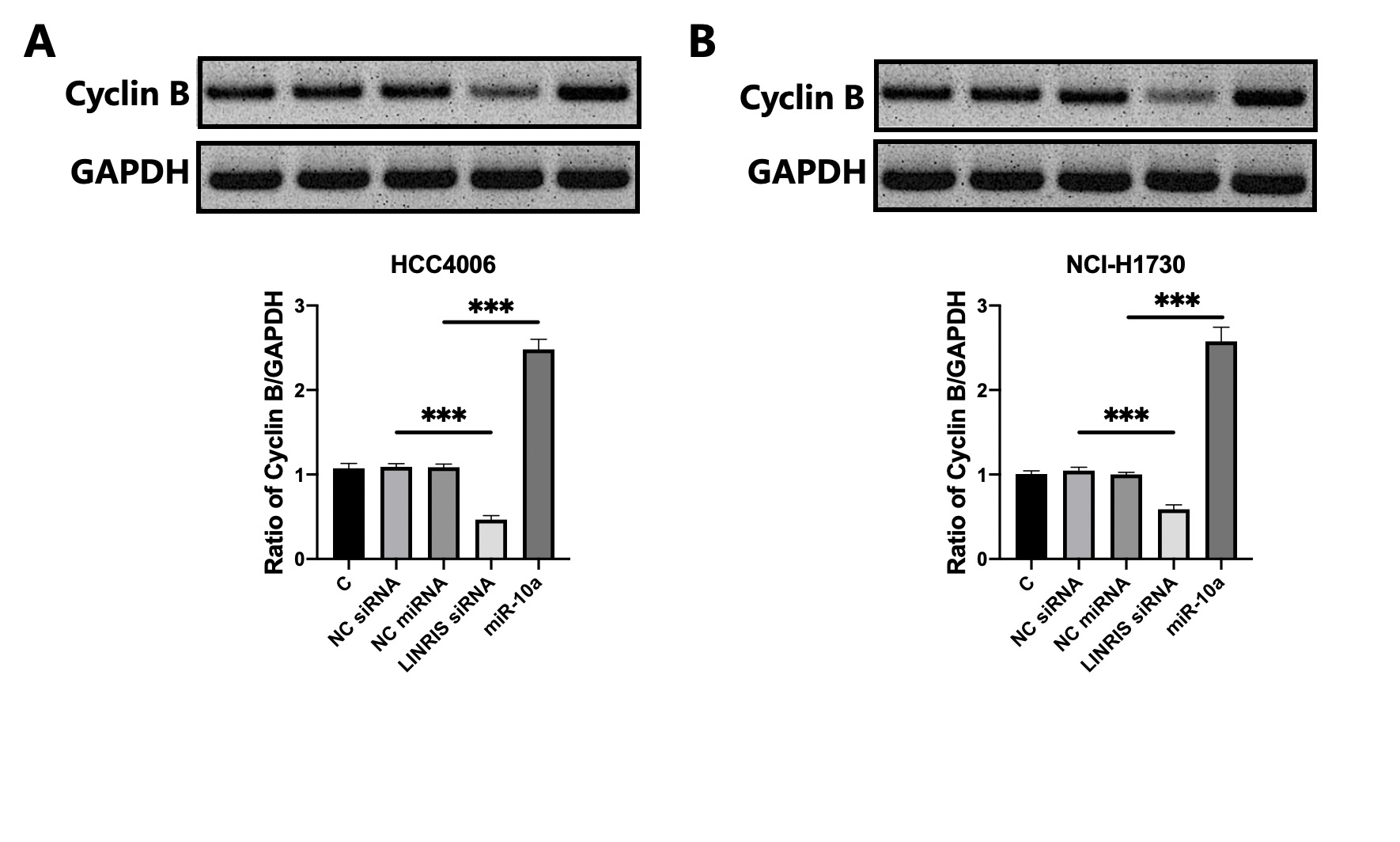

Supplement: Supplemental Material [file KBIE_A_2031672_SM7453.zip › supplementary/Figure S3.tif]

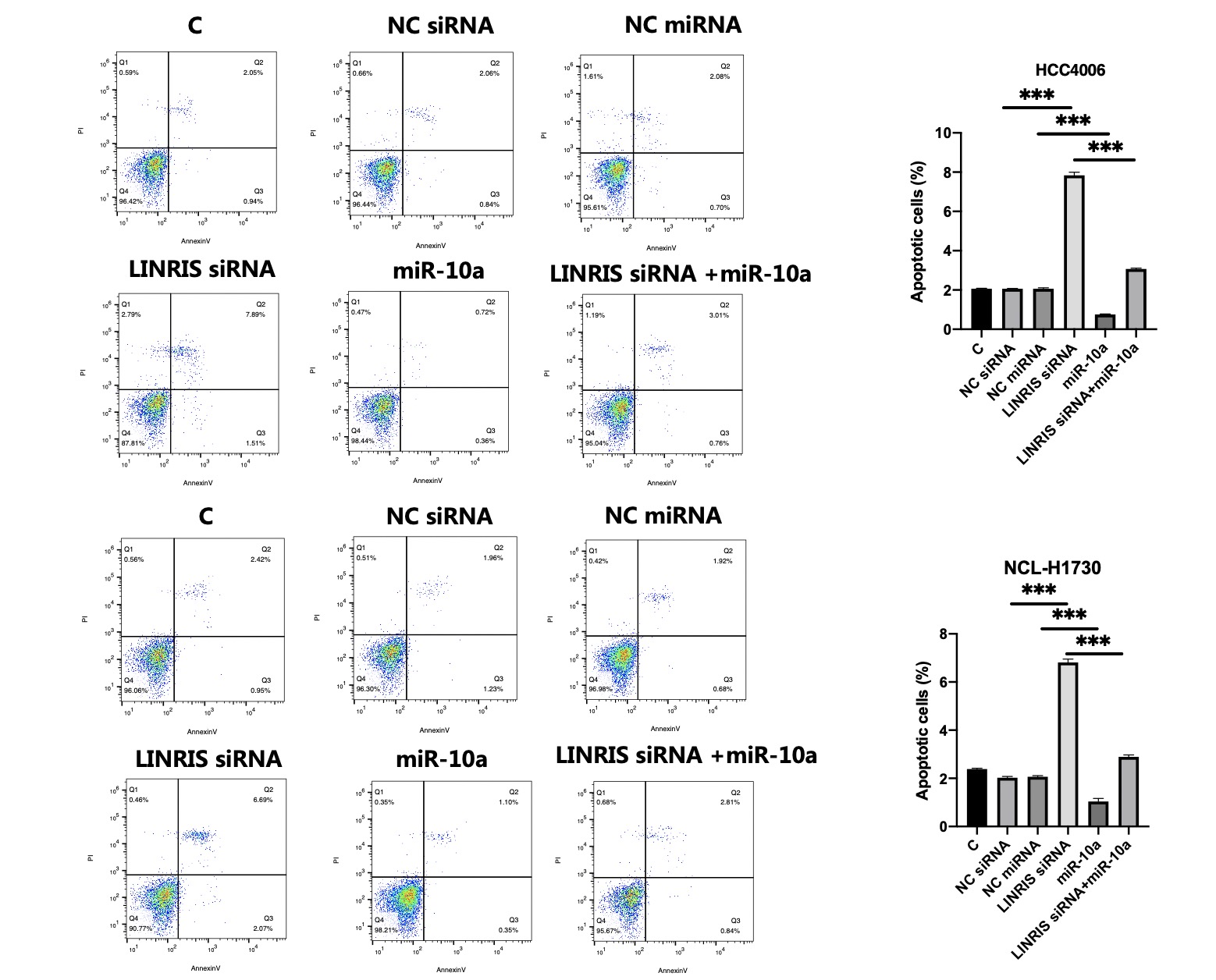

Supplement: Supplemental Material [file KBIE_A_2031672_SM7453.zip › supplementary/Figure S4.tif]
